# Comparative Static and Dynamic Analyses of Solvents for Removal of Asphaltene and Wax Deposits above- and below-Surface at an Iranian Carbonate Oil Field

**DOI:** 10.1021/acsomega.3c03149

**Published:** 2023-07-06

**Authors:** Milad Norouzpour, Amin Azdarpour, Rafael M. Santos, Ali Esfandiarian, Moein Nabipour, Erfan Mohammadian, Abbas Khaksar Manshad, Alireza Keshavarz

**Affiliations:** † Department of Petroleum Engineering, Marvdasht Branch, Islamic Azad University, Marvdasht 73711-13119, Iran; ‡ School of Engineering, University of Guelph, Guelph, Ontario N1G 2W1, Canada; § Key Laboratory of Continental Shale Hydrocarbon Accumulation and Efficient Development, Northeast Petroleum University, Daqing 163318, Heilongjiang, China; ∥ Department of Petroleum Engineering, Abadan Faculty of Petroleum Engineering, Petroleum University of Technology (PUT), Abadan 49658-15879, Iran; ⊥ Petroleum Engineering Discipline, School of Engineering, Edith Cowan University, 270 Joondalup Drive, Joondalup, WA 6027, Australia; # Centre for Sustainable Energy and Resources, Edith Cowan University, Joondalup, WA 6027, Australia

## Abstract

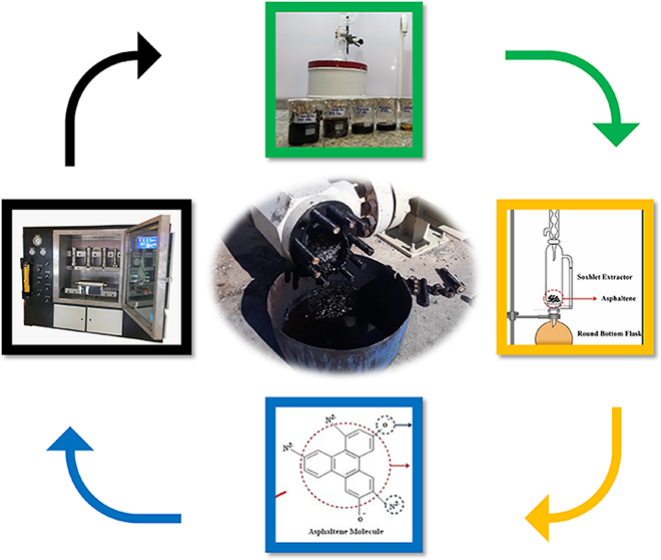

During production from oil wells, the deposition of asphaltene and wax at surface facilities and porous media is one of the major operational challenges. The crude oil production rate is significantly reduced due to asphaltene deposition inside the reservoir. In addition, the deposition of these solids inside the surface facilities is costly to oil companies. In this study, the efficiency of different solvents in dissolving asphaltene and wax was investigated through static and dynamic tests. The analysis of solid deposits from the surface choke of one of the Iranian carbonate oil fields showed that they consisted of 41.3 wt % asphaltene, and the balance was predominantly wax. In addition, the asphaltenes obtained from the surface choke solid deposits had a more complex structure than that of asphaltenes extracted from the crude oil itself. The static tests showed that xylene, toluene, gasoline, kerosene, and gas condensate had the highest efficiencies in dissolving solid deposits; conversely, diesel had a negative impact on dissolving solid deposits. Static tests on pure asphaltene showed that, among the tested solvents, gas condensate and diesel had a negative effect on the solubility of asphaltene. The dynamic core flooding results showed that asphaltene deposition inside the cores reduced the permeability by 79–91%. Among the tested solvents, xylene, gasoline, and kerosene resulted in the highest efficacy in restoring the damaged permeability, and higher efficiency was obtained with an equivalent solvent injection rate of 1 bbl/min versus 3 bbl/min.

## 1 Introduction

Crude oil is a mixture of various quantities and forms of organic components such as resin, wax, and asphaltene. Asphaltene is the heaviest and most polar compound of crude oil.^1−3^ Asphaltene is usually insoluble in normal alkanes and soluble in aromatics.^4−6^ Any disturbance in the thermodynamic equilibrium of the crude oil results in asphaltene precipitation and deposition.^7,8^ Changes in pressure and temperature, gas injection, acidizing, and well stimulation are the significant causes of asphaltene deposition.^9−12^ On the other hand, wax is the paraffinic fraction of crude oil, where its solubility decreases by decreasing the temperature.^13−15^ As the temperature falls below the cloud point or wax appearing temperature (WAT), the paraffin forms a high-molecular-weight solid wax. Asphaltenes are possible deposits inside the reservoir, near the wellbore region, inside the tubing string, and at the surface facilities; however, wax is rarely deposited inside the reservoir and near the wellbore region as it usually deposits in downstream sections from tubing to surface facilities.^16−18^ Permeability reduction is the significant consequence of asphaltene and wax deposition inside the reservoir, which results in a considerable decline in production rate.^19−21^

Asphaltene and wax removal from surface facilities and reservoirs is of great economic interest to production companies. The commonly practiced methods are mechanical, chemical, and thermal treatments.^22−25^ Solvent wash is one of the most common asphaltene and wax removal methods, where the injected solvent redissolves these deposits for a while. Different solvents derived from renewable sources, aromatic and aliphatic solvents, cosolvents, and polymeric dispersants have recently been used for asphaltene and wax removal.^26−28^ Although many solvents have been used for this purpose, understating the nature of the deposits and then selecting the best solvent are of great importance.

### 1.1 Concept of This Study

The “X” oil field in southern Iran has been producing nearly 15 000 bbl/day; however, the production rate has recently declined to about 6000 bbl/day. The primary reason for this decline is due to the deposition of organic compounds, mainly asphaltene and wax. The results of static tests in various wells showed an abnormally high-pressure drop in the reservoir due to discontinuity in the flow regime because of permeability blockage. In addition, the well test results show a positive skin effect near the wellbore region. These two results prove that reservoir is damaged because of the deposition of these organic compounds. In addition, it was observed during workover activities that the downhole tools face some restrictions in reaching the bottom of the well, which is due to the deposition of organic compounds in the well column. Moreover, the deposition of these organic compounds is observed at the surface facilities.

**Figure 1:**
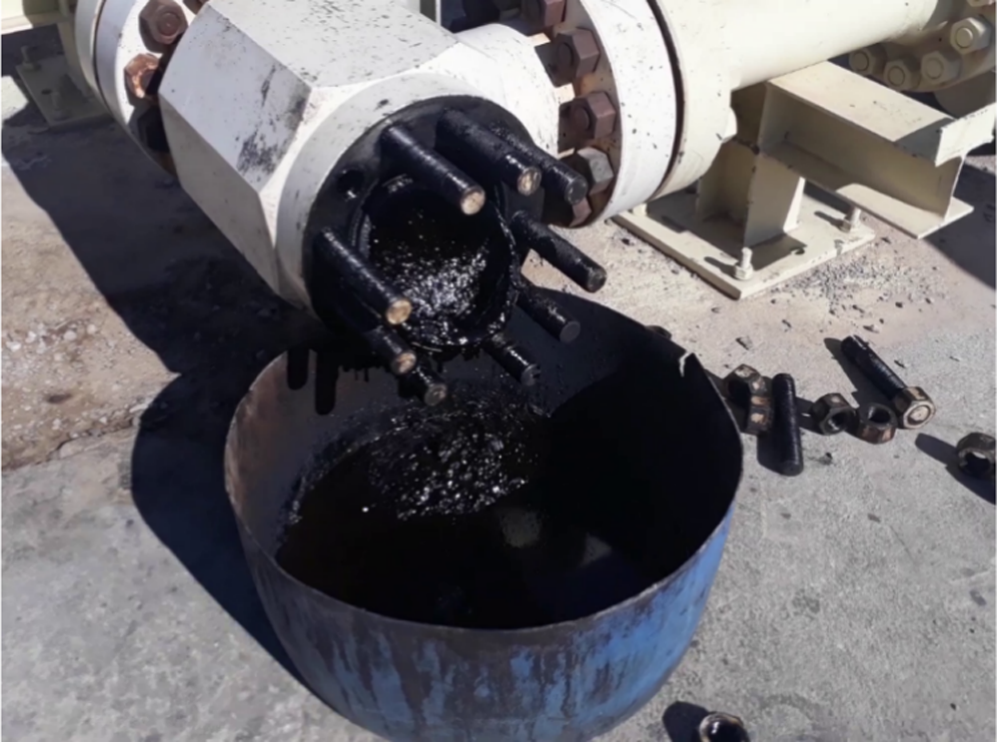
Solid deposits accumulated behind the surface choke.

Figure 1 shows these solid deposits accumulated behind the surface choke of one of the wells, where oil flow has dropped significantly. There remains no doubt that the deposition of organic compounds has resulted in a loss of oil production rate by damaging the reservoir permeability and restricting the oil flow. Thus, this study was performed to investigate the nature of organic compounds accumulated at the surface facilities and to propose practical solutions to eliminate these solids and retrieve the damaged permeability. In this regard, a comprehensive solvent-based study was conducted to explore the effectiveness of different types of solvents in dissolving wax and asphaltene deposits. The significant difference between the current study and the published articles in the literature is that this study focuses on a broad range of solvents, including aliphatics and aromatic solvents, as well as their mixture to dissolve wax and asphaltene deposits. In addition, the injection rate as one of the most critical factors during chemical injection was explored, and the efficiency of each solvent with low and high injection rates was investigated.

## 2 Materials and Methods

### 2.1 Fluids

The efficiency of solvents in dissolving the solid deposits was investigated using aliphatic and aromatic solvents such as xylene, toluene, gasoline, kerosene, gas condensate, and diesel. Crude oil and formation brine were collected from the Sarvak formation in one of the Iranian carbonate oil fields. In addition, cyclohexane was used for measuring the base permeability. The API of the crude oil used in this study was about 32.8. The SARA analysis of the crude oil was as follows: 68.7% saturate, 24.9% aromatics, 5.5% resin, and 0.9% asphaltene. The colloidal instability index (CII) is a measure of asphaltene stability determination (eq 1), where asphaltene is unstable for CII > 0.9. According to this equation, the CII of the crude oil used in this study is 2.29, which clearly shows that the asphaltene is very unstable. The results of the composition analysis of the crude oil used in this investigation are shown in Table S1. In addition, the total dissolved solids (TDSs) of the formation brine were about 200 000 ppm
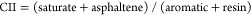
1

### 2.2 Rock Properties

The used carbonate core samples in this study were taken from outcrops of the Sarvak formation, and rock properties were determined using X-ray diffraction (XRD) analysis and ζ-potential measurements. A D8 Bruker XRD (made by Germany) was used for determining the XRD pattern of the rock sample. The XRD analysis of the carbonate sample showed that it is mainly composed of dolomite. The XRD pattern of the carbonate core sample is shown in Figure S1a.

A microprocessor-equipped SZ-100 (Horiba Institute, Japan) zeta-meter was employed for streaming potential measurements. Utilizing the Smoluchowski formula as shown by eq 2, the device automatically measures the electrophoretic mobility of the particles and translates it into ζ-potential
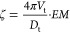
2where *V*_t_ is the viscosity of the suspending liquid, *D*_t_ represents the dielectric constant, EM corresponds to the electrophoretic mobility, and ζ is the ζ-potential.^29^ A sample of 0.3 g dolomite rock powder was combined with 100 mL of distilled water that contained the appropriate electrolyte at 25 ± 2 °C for 48 h to determine a point of zero charge (PZC = 6.7). The ζ-potential values versus pH are shown in Figure S1b.

### 2.3 Solid Deposits (Asphaltene and Wax)

In this study, solid deposits are divided into four parts, including asphaltene extracted from crude oil (AECO), wax extracted from crude oil (WECO), solid deposits accumulated behind the surface choke (SC), and asphaltene extracted from SC (AESC). The accumulation of these SCs has reduced the oil flow rate significantly. These solid deposits are mainly composed of asphaltene and wax, as explained in the following sections. First, the oil and heptane in a volume ratio of 1–40 were stirred for 24 h to precipitate the asphaltene and wax. Then, the heptane and crude oil solution was strained through the Whatman filter paper. In the next step, precipitated solid deposits were washed with hot heptane to separate the asphaltene and wax.^30^ The schematic diagram of the extraction process schematically by the modified IP-143 (ASTM D6560) procedure is shown in Figure S2.

### 2.4 Core Samples

As mentioned before, the used carbonate core samples in this investigation were taken from outcrops of the Sarvak formation. The porosity of samples was measured using a helium porosity setup (Fars EOR Tech), and the absolute permeability was measured using a GasPerm (Fars EOR Tech) setup. The porosity of samples was between 11 and 16%, and the permeability of samples was between 5.83 and 11.83 mD. The diameter of core samples was 1.9 cm, while the length of samples was between 6.73 and 6.80 cm. The physical properties of the core samples utilized in this study are presented in Table S2.

### 2.5 Determination of Injection Rate during Core Flood Experiments

In order to investigate the effect of injection rate on the efficiency of solvents, the constant injection rate could not be used for all of the cores because the porosity and length of each core were different. Thus, the concept of fluid velocity inside porous media was utilized. In this method, the actual velocity of the fluid inside the reservoir was calculated as *V* = *Q*/*A*. Then, a back-calculation was made using the calculated velocity, and the injection rate to the cores was calculated in terms of cm^3^/min. Solvent injection to the reservoir is carried out using coiled tubing, where a standard injection rate of 1–3 bbl/min could be applied. Thus, in this study, the minimum and the maximum injection rates of 1 and 3 bbl/min were considered accordingly. The summary of calculated injection rates for each core sample is presented in Table S3.

### 2.6 Static Tests

The static bottle tests were carried out using solid deposits collected from the surface choke. In these experiments, 1 g of solid deposits was weighted and poured into a beaker. After that, different volumes (10–100 mL) of solvents (xylene, toluene, gasoline, diesel, gas condensates, and kerosene) were added to the beaker. Then, a different retention time of 1–10 days was applied to solvents, and solutions were filtered afterward. Finally, the weight of solids deposited on the filter paper was measured, and the efficiency of each solvent was calculated.

### 2.7 Core Flood Experiments

The dynamic tests were carried out using the core flood setup shown in Figure S3.^31^ The core flood setup used in these experiments consists of two HPLC pumps, three accumulators, a differential pressure measurement, a core holder, an oven for providing constant heat, a hand pump for proving confining pressure, and a backpressure regulator. The differential pressure was recorded during the experiments, and the permeability was calculated using eq 3. In this equation, *K* is in mD, *q* is in cm^3^/min, *A* is in cm^2^, μ is in cP, Δ*P* is in kPa, and *L* is in cm^32−34^
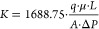
3The core flood procedure consists of eight major steps:

(i) Core samples were cleaned, dried, and vacuumed for about 24 h.
(ii) Each core was saturated with formation brine at 2000 psi for about 7 days.
(iii) A total of 5 PVs of formation brine was injected into the core.
(iv) Injection of cyclohexane to the core and measuring the initial permeability at the connate water saturation. In this step, 20 PVs were injected to determine cyclohexane permeability.
(v) Injection of crude oil into the cores. In this step, 20 PVs of crude oil were injected into the cores.
(vi) A total of 20 PVs of cyclohexane was injected, and the cyclohexane permeability was measured. The calculated permeability in this section depicts the damaged permeability due to asphaltene deposition inside the cores.
(vii) Different solvents, including xylene, kerosene, and gasoline, and their mixture with gas condensate were prepared. A 50–50% volumetric ratio was used to prepare the mixed solvents. Then, each solvent was injected into the core with the calculated injection rates. The retention time of 1–3 days was applied depending on the definition of each test.
(viii) A total of 20 PVs of cyclohexane was injected, and the cyclohexane permeability was measured. The calculated permeability in this section depicts the improved permeability by solvent injection.

It is worth mentioning that the reservoir conditions were applied in these experiments, where a temperature of 80 °C, a backpressure of 4000 psi, and a confining pressure of 4500 psi were used in all experiments. The summary of the procedure of core flood experiments conducted in this study is shown in Figure S4. It is worth mentioning that the reason for cyclohexane in this study is that this pure hydrocarbon does not affect in situ asphaltenes by causing extra deposition or dissolving. Therefore, it could be used as a reference case for comparison during flooding experiments.

### 2.8 Fourier Transform Infrared (FT-IR) Spectroscopy

The extracted mixtures were evaluated using FT-IR analysis between 400 and 4000 cm^–1^ on a Perkin Elmer RX1 infrared (IR) spectrophotometer utilizing KBr pellet methods. The spectra were interpreted by IR-Pal V2.0 FT-IR spectra analysis software (Dr. Wolf van Heeswijk) and previous research.

## 3 Results and Discussion

### 3.1 Asphaltene Content of the Solid Deposits

Initially, 20 g of solid deposits taken from behind the surface choke was measured, and the asphaltene content was determined. This experiment was repeated three times, and finally, the asphaltene content of the solid deposits was measured by averaging the results. Table 1 presents the weight of samples before and after n-heptane addition and the asphaltene contents. As presented, the average asphaltene content of the solid deposits is about 41.34 wt %. The remaining percentage of the solid deposits is wax, as no evidence of mineral deposits was detected during the filtration process. These results suggest that wax and asphaltene deposit at surface facilities, such as surface chokes.

**Table 1:** Asphaltene Content of Solid Deposits

the initial weight of solid deposits (g)	the final weight of solid deposits (g)	asphaltene content (wt %)	average asphaltene content (wt %)
20	8.412	42.06	41.34
20	7.761	38.80	
20	8.631	43.51	

### 3.2 Static Tests on the Solid Deposits Containing Wax and Asphaltene

**Figure 2:**
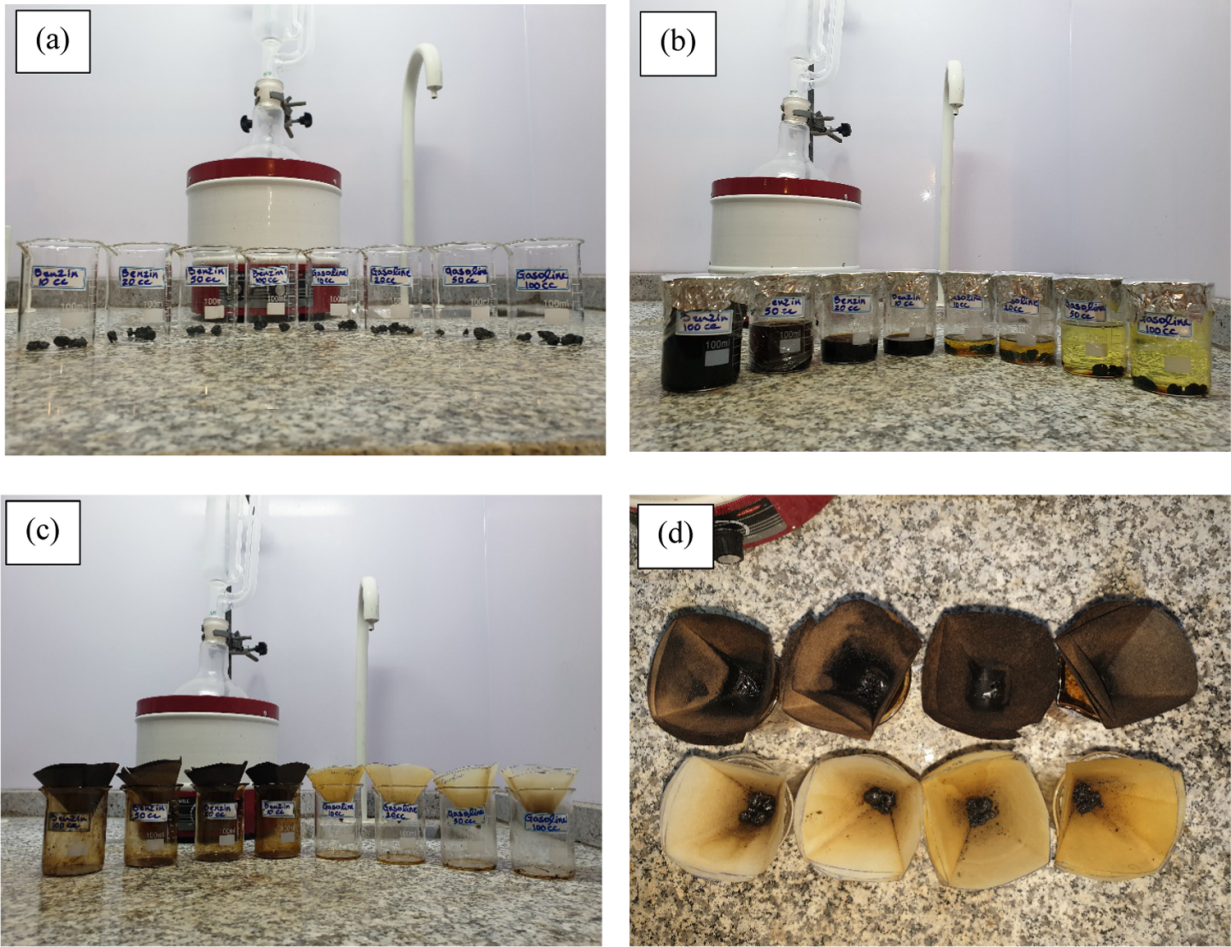
Images of solubility measurement tests with different solvents: (a) images of 1 g of solid deposits in a beaker at the start of experiments; (b) images of different volumes of solvents added to each beaker at the start of experiments; (c) images of different solutions during filtration after 72 h; and (d) images of solid residues after filtration.

In this section, different static bottle tests were performed, and the efficiency of each solvent in dissolving 1 g of solid deposits (wax and asphaltene) was determined. Figure 2a presents the images of 1 g of solid deposits in a beaker. Figure 2b presents images of different volumes of solvents added to each beaker. Figure 2c presents images of different solutions during filtration after 72 h, and Figure 2d presents solid residues after filtration. The weight of solid deposits on the filter paper was used for calculating the efficiency of each solvent in dissolving 1 g of solid deposits accordingly.

#### 3.2.1 Screening of the Solvents

**Figure 3:**
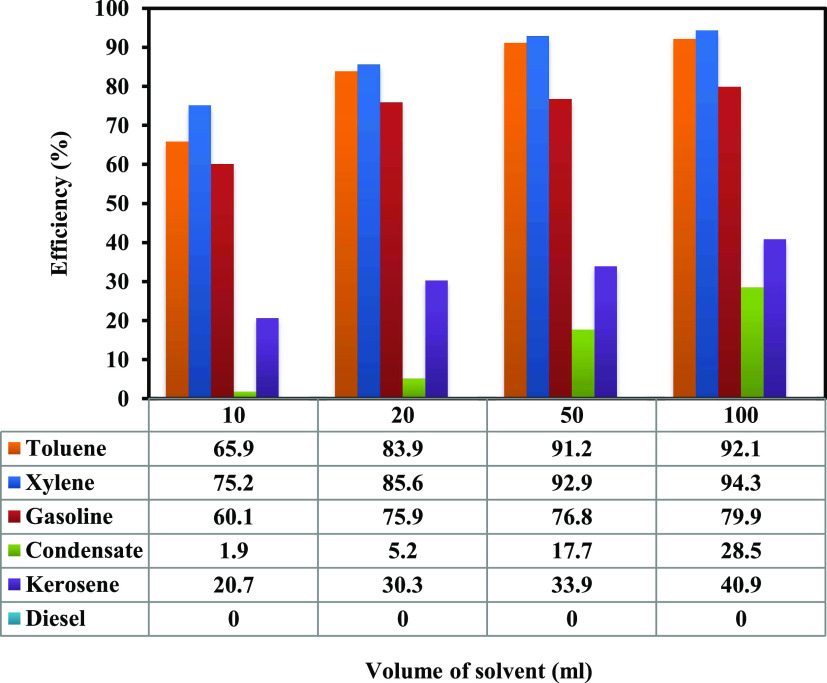
Effect of solvent volume on the efficiency of solvents.

Figure 3 presents the efficiency of different solvents in dissolving solid deposits with a variable volume of each solvent at a constant reaction time of 72 h. It is worth mentioning that solid deposits containing wax and asphaltene were used in this section. Experimental results showed three different behaviors with the used solvents; the first group of solvents was xylene, toluene, and gasoline, which showed similar results. The second group was diesel and gas condensate, which showed similar results. On the other hand, diesel showed a different behavior than other solvents. These findings agree with the literature results.^33,35,36^

Among the tested solvents, the maximum efficiency was for xylene, toluene, gasoline, kerosene, and gas condensate. All of the tested solvents (except diesel) responded well by increasing the solvent volume from 10 to 100 mL. The lowest efficiency of each solvent was achieved when 10 mL of the solvent was used, and the efficiency reached the maximum value when the solvent volume increased to 100 mL. The efficiency of 94.3, 92.1, 79.9, 40.9, and 28.5% was achieved for xylene, toluene, gasoline, kerosene, and gas condensate, respectively, when the volume of 100 mL was used for each solvent. On the other hand, the efficiency of diesel was zero with all of the tested volumes. The weight of solid deposits increased when the diesel volume was increased, resulting in negative diesel performance. The weight of 1 g of solid deposits increased to 1.235, 1.273, 1.334, and 1.407 g after adding 10, 20, 50, and 100 mL of diesel, respectively.

Xylene and toluene are potent aromatic solvents for dissolving organic compounds, especially asphaltene; however, toluene is less effective than xylene. These solvents can dissolve a significant fraction of asphaltene even at low volumes.^22,37−39^ The experiments’ results confirmed these solvents’ applicability in dissolving organic deposits. On the other hand, gasoline, diesel, gas condensates, and kerosene are aliphatic solvents. The aliphatic solvents are good candidates for dissolving paraffinic deposits such as wax; however, they are ineffective for asphaltene removal.^39−43^ Among the tested aliphatic solvents in this study, gasoline showed better results than the others. Gasoline is mainly composed of aliphatics; however, some impurities (mainly aromatics) are added to its composition to increase the octane number. These aromatic components in gasoline give unique gasoline characteristics in dissolving the asphaltene deposits. Kerosene and gas condensates are hydrocarbon-based aliphatics, which are relatively purer than gasoline in terms of added aromatics. Thus, they are less effective than gasoline in dissolving solid deposits. On the other hand, diesel is known to be the heaviest aliphatic, with a significant number of impurities in its composition.^42−46^ The presence of these impurities results in the formation of new bonds between diesel molecules and asphaltene deposits, resulting in the increase of the weight of asphaltene deposits and giving negative efficiency to diesel.

#### 3.2.2 Efficiency of Aliphatic Solvents

Aromatic solvents such as xylene and toluene are known to be very effective in removing asphaltene deposits; however, safety issues and their high price are significant obstacles to be considered. The role of aliphatic solvents such as gasoline, gas condensates, and kerosene as cheaper, widely available, and less risky solvents to dissolve solid deposits is important. Thus, static tests using the abovementioned aliphatic solvents were performed, and the efficiency of each solvent in dissolving 1 g of solid deposits was determined accordingly, as presented in Table 2.

**Table 2:** Efficiency of Aliphatic Solvents in Dissolving 1 g of Solid Deposits

solvent	the volume of solvents (mL)	retention time (days)	weight of solid deposits after filtration (g)	efficiency of solvent (%)
gasoline	20	1	0.375	62.5
gasoline	20	3	0.241	75.9
gasoline	20	7	0.232	76.8
gasoline	20	10	0.225	77.5
gasoline	100	1	0.325	67.5
gasoline	100	3	0.201	79.9
gasoline	100	7	0.184	81.6
gasoline	100	10	0.169	83.1
kerosene	20	1	0.703	29.7
kerosene	20	3	0.697	30.3
kerosene	20	7	0.685	31.5
kerosene	20	10	0.679	32.1
kerosene	100	1	0.613	38.7
kerosene	100	3	0.591	40.9
kerosene	100	7	0.579	42.1
kerosene	100	10	0.571	42.9
gas condensate	20	1	0.976	2.4
gas condensate	20	3	0.948	5.2
gas condensate	20	7	0.937	6.3
gas condensate	20	10	0.932	6.8
gas condensate	100	1	0.894	10.6
gas condensate	100	3	0.715	28.5
gas condensate	100	7	0.701	29.9
gas condensate	100	10	0.683	31.7

The static test results showed that all three tested solvents are affected by the retention time and the solvent volume. Increasing the retention time from 1 to 10 days and the volume of solvent from 20 to 100 mL increased the efficiency of solvents. The best performance of each solvent was achieved when a retention time of 10 days and a volume of 100 mL were used, where this value was 83.1, 42.9, and 31.7% for gasoline, kerosene, and gas condensate, respectively. As was expected, the presence of aromatics in the gasoline content increased the solubility of solid deposits compared to the other two solvents. These findings are in good agreement with the results in the literature.^47−51^

#### 3.2.3 Efficiency of the Mixed Solvents

**Figure 4:**
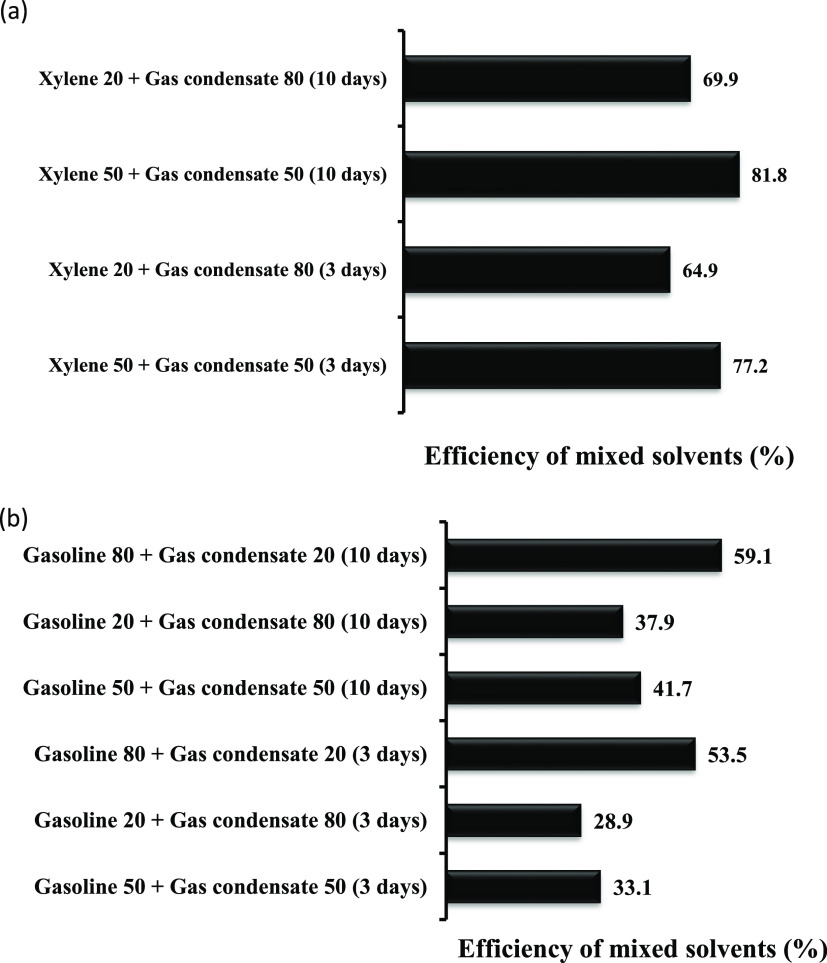
(a) Efficiency of the mixture of xylene and gas condensate in dissolving solid deposits; (b) efficiency of the mixture of gasoline and gas condensate in dissolving solid deposits.

Due to the availability of aliphatic solvents and the toxic nature of the aromatic solvents, the efficiency of mixed solvents prepared by different proportions of these mixtures in dissolving solid deposits was examined. In the first part, different volumetric ratios of xylene and gas condensate were mixed, and the efficiency of the mixed solvent was investigated, as shown in Figure 4a. In the second part, the efficiency of mixed solvents prepared by different volumetric ratios of gasoline and gas condensate was determined, as presented in Figure 4b. In these experiments, the efficiency of solvents was measured after 3 and 10 days.

As shown in Figure 4a, the maximum efficiency of 81.8% is achieved after 10 days when 50 vol % of xylene is mixed with 50 vol % of gas condensate. The minimum efficiency of 64.9% was achieved after 3 days when only 20 vol % of xylene was mixed with gas condensate. These results suggest that increasing the retention time from 3 to 10 days and increasing the volume of xylene from 20 to 50 vol % are effective in increasing the efficiency of the mixed solvent. On the other hand, Figure 4b presents the efficiency of mixed solvents prepared with gasoline and gas condensate. The maximum efficiency was achieved after 10 days of retention time when 80 vol % of gasoline was mixed with 20 vol % of gas condensate, and the minimum efficiency was achieved after 3 days of retention time when 20 vol % of gasoline was mixed with 80 vol % of gas condensate. The efficiency of 59.1 and 28.9% was achieved as the maximum and minimum, respectively. These results suggest that increasing the volume of gasoline had a positive effect on the efficiency of the mixed solvent and resulted in an increase in efficiency. In addition, increasing the retention time from 3 to 10 days also increased the efficiency of the mixed solvent. Table 3 presents the effects of increasing the retention time and volume of xylene on the efficiency of mixed solvents. At a constant volume of xylene, increasing the retention time from 3 to 10 days increased the efficiency by 4.2 and 5%. On the other hand, increasing the volume of xylene from 20 to 50 mL at a constant retention time increased the efficiency by 11.5 and 12.3%. These results suggest that mixed solvents prepared by xylene and gas condensate are more affected by the volume of xylene than the retention time. Increasing the volume of xylene increases the aromatics volume, thus dissolving more solid deposits.^39,41,43^ In addition, mixed solvents prepared with gasoline and gas condensate were also more affected by the volume of the gasoline than retention time, as shown in Table 4. At a constant retention time, increasing the volume of gasoline from 20 to 80 mL increased the efficiency by 21.2 and 24.6%, and at a constant volume of gasoline, the efficiency increased by 9% and 5.6% by increasing the retention time from 3 to 10 days. These results also suggest that mixed solvents prepared with gasoline and gas condensate are more sensitive to the volume of gasoline than the retention time. The volume of aromatics present in gasoline increases with increasing the volume of gasoline; thus, the efficiency of the mixed solvents to dissolve solid deposits is increased.

**Table 3:** Comparison of the Effects of Retention Time and Volume of Xylene on the Efficiency of the Mixed Solvent

increasing the retention time from 3 to 10 days		increasing the volume of xylene from 20 to 50 mL	
volume of xylene, 50 mL	volume of xylene, 20 mL	retention time of 10 days	retention time of 3 days
4.2%	5%	11.5%	12.3%

**Table 4:** Comparison of the Effects of Retention Time and Volume of Gasoline on the Efficiency of the Mixed Solvent

increasing the retention time from 3 to 10 days		increasing the volume of gasoline from 20 to 80 mL	
volume of gasoline, 20 mL	volume of gasoline, 80 mL	retention time of 10 days	retention time of 3 days
9%	5.6%	21.2%	24.6%

#### 3.2.4 Efficiency of the Solvents in Dissolving Pure Asphaltene

In this part, pure asphaltene was extracted from solid deposits using the previous method. Then, the efficiency of different aromatic and aliphatic solvents in dissolving 1 g of asphaltene was determined at a retention time of 7 days, and the results are presented in Table 5. The experimental results showed that xylene, gasoline, and kerosene have the highest efficiency in dissolving asphaltene. The dissolving efficiency of 95.8, 80.6, and 48.2% was achieved with 20 mL of xylene, gasoline, and gas condensate, respectively. These values increased to 100, 85.6, and 50.7% by increasing the volume of solvents to 100 mL, respectively. On the other hand, gas condensate and diesel showed different behavior in dissolving asphaltene. The weight of asphaltene increased with the addition of gas condensate and diesel, as shown in Table 5. In addition, this increase in the weight of asphaltene was in a direct relationship with the volume of solvent, where the higher the volume of solvent, the higher the weight of remaining asphaltene after the experiments.

**Table 5:** Efficiency of Different Solvents to Dissolve 1 g of Asphaltene in 7 Days

solvent	gasoline	xylene	kerosene	gas condensate	diesel
20 mL of Solvents					
weight of the remaining asphaltene after the experiment (g)	0.194	0.042	0.518	1.039	1.273
the efficiency of solvent (%)	80.6	95.8	48.2	0	0
50 mL of Solvents					
weight of the remaining asphaltene after the experiment (g)	0.144	0.000	0.493	1.291	1.313
efficiency of solvent (%)	85.6	100	50.7	0	0

**Figure 5:**
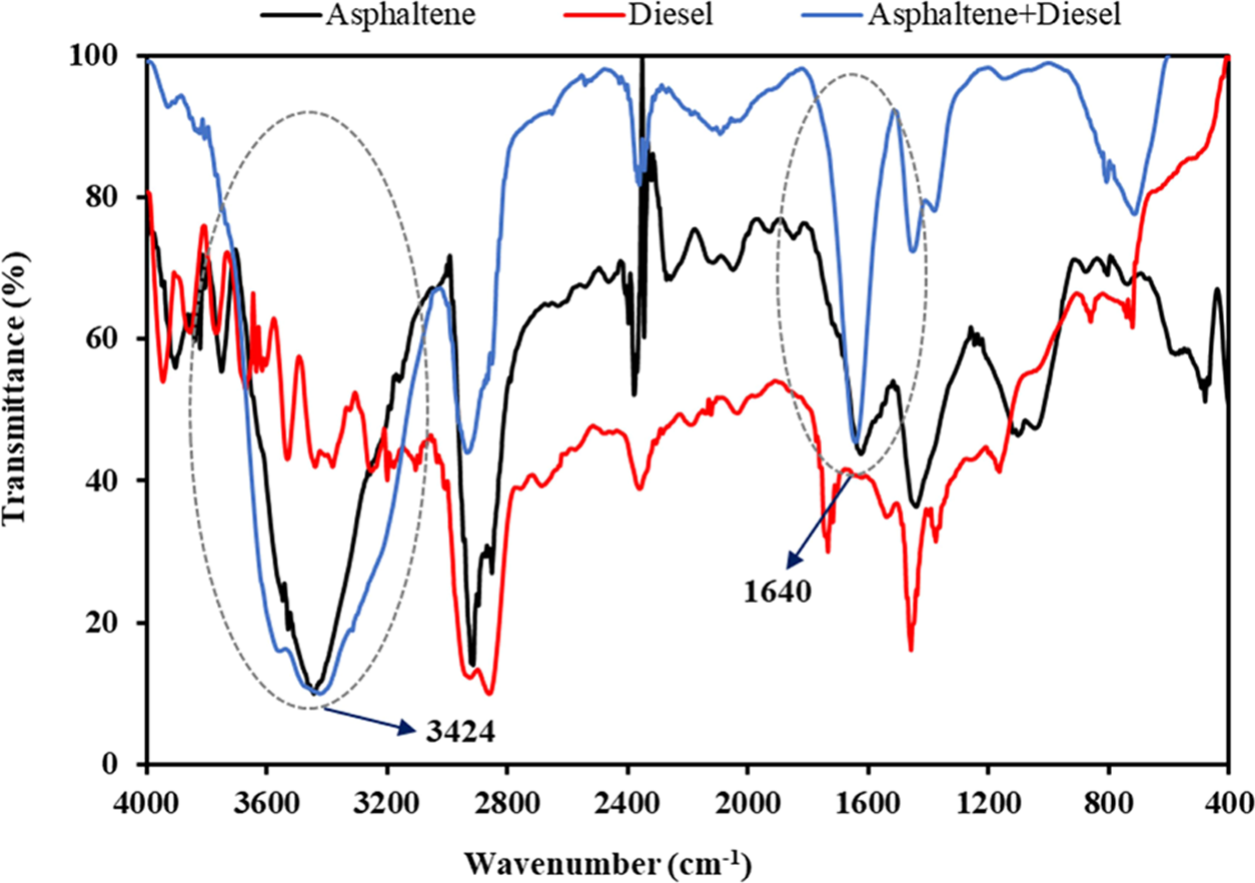
FT-IR analysis of asphaltene, diesel, and asphaltene + diesel samples; circled areas highlight regions of O–H and N–H bonds.

Aliphatic solvents are not potentially perfect solvents for dissolving asphaltene. Instead, they perform well in dissolving wax.^37,40,41,43^ In addition, asphaltene is a polar compound of crude oil with a high affinity for adsorption. Figure 5 presents the FT-IR analysis of asphaltene, diesel, and asphaltene+diesel samples. As shown in this figure, the FT-IR curve of the asphaltene + diesel sample is not completely matched with the asphaltene and diesel curves. By comparing the asphaltene and asphaltene+diesel curves, two broader and more intensive N–H and O–H peaks are observed at the 1640 and 3424 cm^–1^, respectively, due to creating new hydrogen bonds between asphaltene and diesel molecules for asphaltene+diesel. The higher peak intensity and peak area in the FT-IR spectrum correspond to the greater concentration and stronger bonded, respectively.^20,52−54^ These hydrogen bonds are owing to partial negative and positive charges (δ^–^ and δ^+^) of oxygen and nitrogen atoms that are present in asphaltene molecules with hydrogen atoms of diesel molecules. In other words, the attachment of diesel and gas condensate to the asphaltene molecules is due to the creation of new hydrogen bonds that result from the electronegative oxygen and nitrogen atoms of asphaltene with hydrogen atoms of diesel and gas condensate. Thus, the adsorption of diesel and gas condensate increases the weight of asphaltene. Consequently, the dissolving efficiency of gas condensate and diesel becomes negative, as observed in this study.

### 3.3 Dynamic Tests (Core Flood Experiments) to Dissolve Asphaltene Deposition

#### 3.3.1 Permeability Damage Due to Asphaltene Deposition

The permeability measurement results showed that asphaltene deposition could cause severe damage to the permeability of cores. In these experiments, 20 PVs of crude oil were injected into the core samples and then followed by 20 PVs of *n*-heptane injection and then permeability was measured. The results showed that 79–91% of the permeability was reduced by asphaltene deposition during crude and n-heptane injection. This damage to the core is because of asphaltene deposition inside the cores, as no wax is deposited at this condition. The WAT of the crude oil used in this study is about 64 °C, suggesting that no wax is deposited at 80 °C.

**Figure 6:**
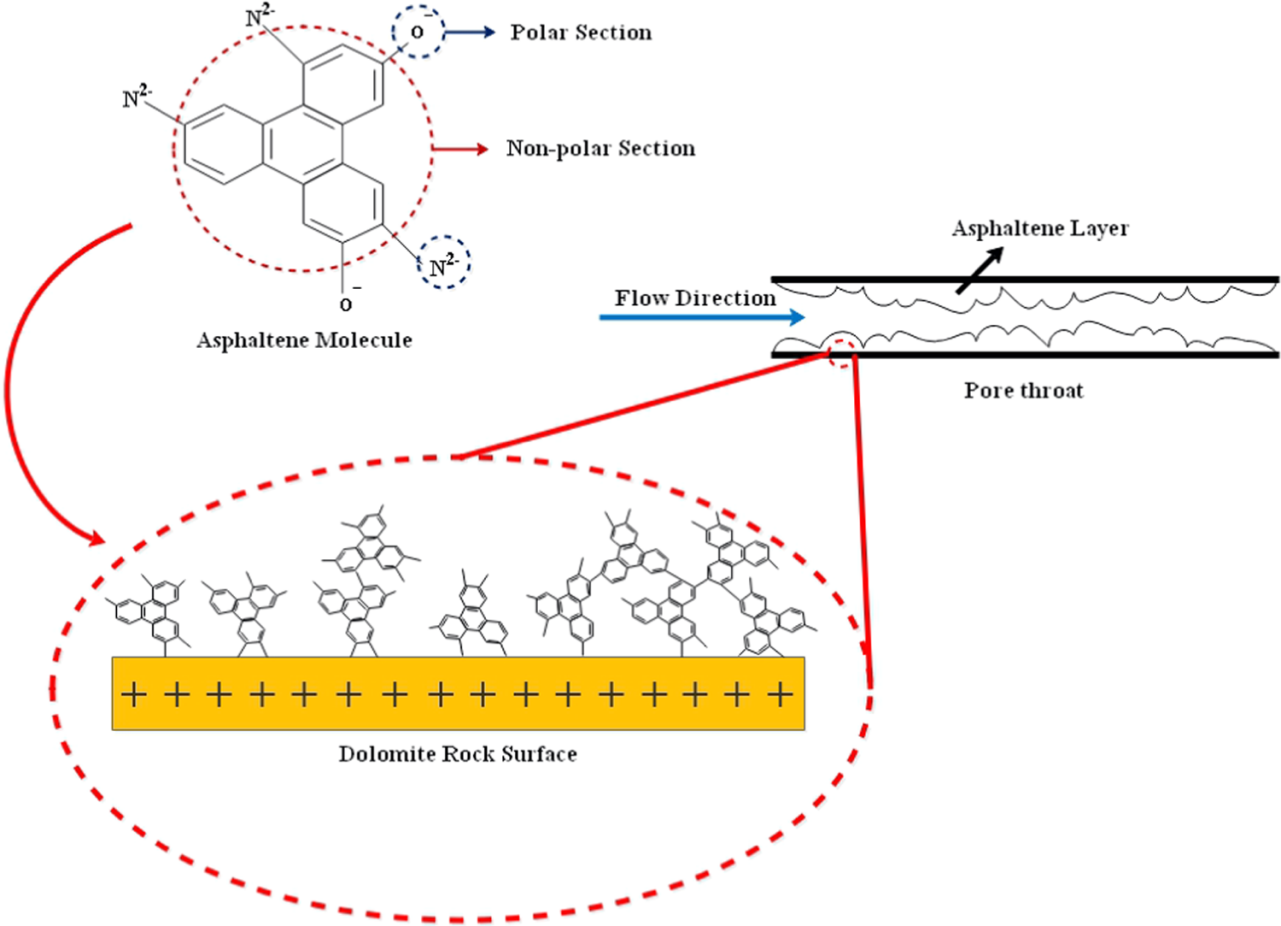
Schematic diagram of permeability reduction by asphaltene adsorption on the dolomite rock surface.

Surface adsorption of asphaltene and pore blocking are the two major mechanisms of permeability reduction by asphaltene deposition. Based on results from ζ-potential tests, the surface charge of dolomite rock is positive when pH is under 6.7 (PZC). Moreover, crude oil is an acidic mixture due to the presence of a large number of fatty acids. Hence, the charge of the rock surface is positive in such an environment. On the other side, asphaltene particles of this specific crude oil have an overall negative charge due to the presence of O–H and N–H bonds in their structures based on FT-IR results. The adsorption mechanism is directly related to the asphaltene content of the crude and surface charge of dolomite rock, where the asphaltene particles are gradually formed asphaltene layers and deposited into the rock surface due to the gravity of opposite charges, causing a reduction in the permeability. Furthermore, asphaltene particles are very heavy and polar and prefer to stick onto the rock surface (solid surface) instead of dispersing into the liquid phase (oleic or aqueous phases) when conditions become thermodynamically unstable for them. On the other hand, pore blocking occurs when asphaltene particles are connected and form a bridge on the pore diameter, increasing the rate of asphaltene accumulation and reducing the core permeability.^55−57^ The reservoir cores used in this study are slightly tight in terms of permeability (5.83–11.83 mD). On the other hand, the asphaltene content of the crude oil used was about 0.9 wt %. Since the asphaltene content of the crude oil is relatively low and the cores’ permeability is very low, it may suggest that pore blocking is the dominant mechanism of permeability reduction in these experiments. Figure 6 shows the mechanism of permeability reduction of porous media by asphaltene precipitation and adsorption on the dolomite rock surface schematically.

#### 3.3.2 Effect of Retention Time on the Efficiency of the Solvent

Initially, three experiments were performed to determine the adequate retention time on the efficiency of the injected solvents. After asphaltene deposition inside the cores and measuring the damaged permeability by cyclohexane, xylene with a low flow rate was injected into the cores and then different retention times of 1, 2, and 3 days were applied at reservoir conditions (4000 psi and 80 °C). Finally, the permeability affected by the solvents was measured using cyclohexane, as presented in Table 6.

**Table 6:** Effect of Retention Time during Low-Rate Injection of Xylene on Improved Permeability

injection rate (cm^3^/min)	retention time (days)	initial permeability (mD)	permeability after damage (mD)	permeability after solvent injection (mD)	damage due to asphaltene deposition (%)	efficiency of the solvent (%)
1.68	1	6.83	1.14	2.19	83	32.06
1.88	2	5.83	1.12	2.19	81	37.56
1.50	3	8.19	1.62	3.19	80	38.95

The permeability results showed that solvent injection to damaged cores effectively improves permeability. The permeability measurement results showed that increasing the retention time from 1 to 2 days was very effective in improving the permeability; however, further increasing the time to 3 days had minimal effect on the efficiency. The minimum efficiency of 32.06% was achieved after 1 day, and it increased to 37.56 and 38.95% by increasing the retention time to 2 and 3 days. Increasing the retention time provides more time for xylene to interact with the asphaltene particles. Thus, permeability increases with retention time; however, this increase is not significant at some point. Hence, the retention time of 2 days was selected as the optimum retention time of solvents in the following experiments.

#### 3.3.3 Effect of Xylene, Gasoline, and Kerosene on Permeability

The mechanism that reduces the permeability of the core sample must be clarified to find a suitable remedy. Hence, in the first step, the interactions between asphaltene and rock must be evaluated using FT-IR analysis and ζ-potential measurements for asphaltene and rock, respectively.

**Figure 7:**
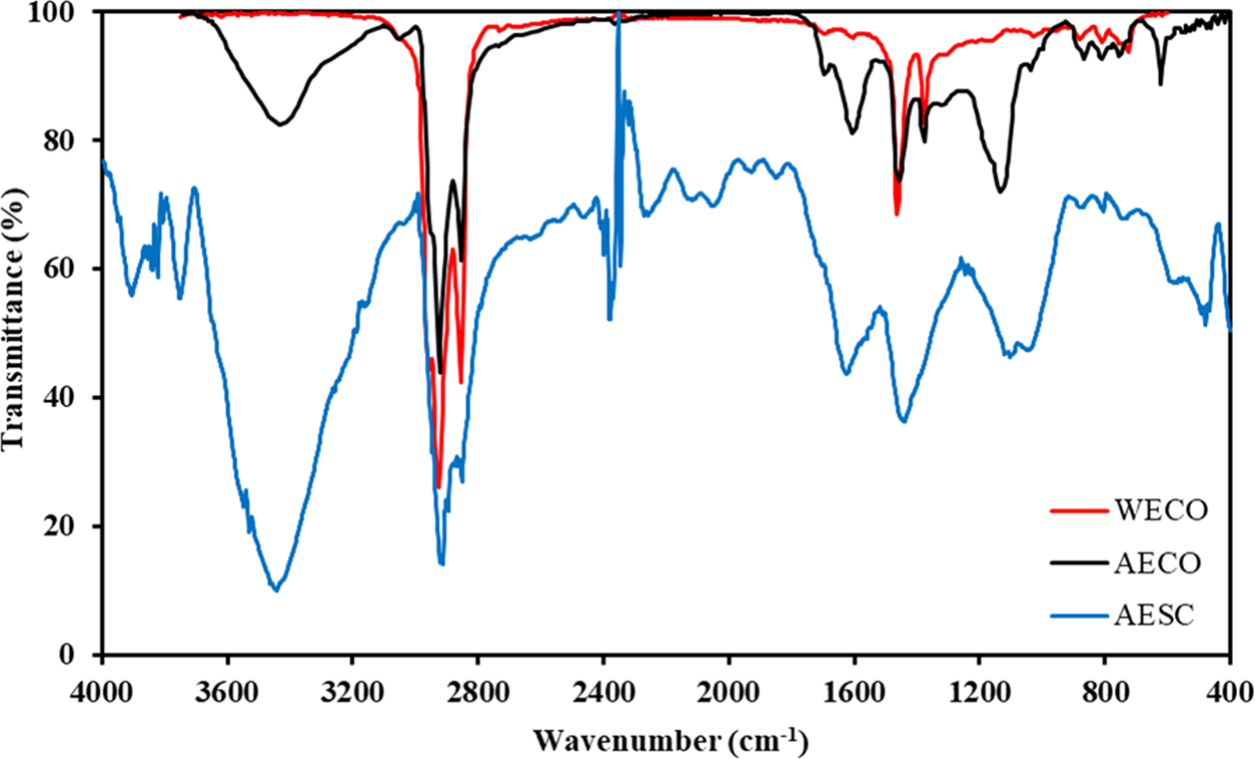
FT-IR analysis of AECO, WECO, and AESC samples.

According to Figure 7 and Table 7, asphaltene extracted from crude oil has a more straightforward structure than asphaltene extracted from solid deposits. It means that the asphaltene structure became more complicated after the deposition and precipitation of asphaltene and wax due to a drop in pressure and temperature from the bottom hole to the chock. By comparing the peaks of the diagrams related to AECO, WECO, and AESC, it is seen that with the simultaneous deposition of asphaltene and wax with each other, some functional groups are added to the asphaltene structure that is not soluble in the normal heptane. Moreover, the higher peak intensity and peak area attributed to the O–H functional group in the FT-IR spectrum of AESC (3424 cm^–1^) than that of AECO (3431 cm^–1^) correspond to the more concentration and stronger hydrogen-bonded, respectively.^58,59^ Hence, functional groups added to the asphaltene structure are not soluble by normal heptane not only for the formation of π–π stacking, charge-transfer interactions, multiple forces, and van der Waals interaction but also the stronger and more hydrogen bonds are formed in AESC.^60^ The IR spectrum at 1800 cm^–1^ corresponds to the C=O stretch of R–CO–Cl that never exists in the AECO and WECO structures.^31^ It shows that after well stimulation by HCl, residual Cl^–^ ions from stimulation operation can enter the asphaltene structure during the production. This hypothesis is confirmed by the presence of a more intense peak at 550 cm^–1^ in the AESC than that of 545 cm^–1^ in AECO, attributed to the C–Cl stretch of R–Cl in alkyl halides. The four unique peaks (2689, 2664, 2410, and 2404 cm^–1^) in the AECO IR spectrum indicate the presence of phosphine and phosphonic acid functional groups that never exist in the AECO and WECO structures.^52^

**Table 7:** Description of the Characteristic Adsorption Bands in the FT-IR of Asphaltene Samples, Including Asphaltene Extracted from Crude Oil (AECO), Wax Extracted from Crude Oil (WECO), and Asphaltene Extracted from Solid Deposits (AESC) Accumulated behind the Surface Choke and Their Related Functional Groups

characteristic adsorption bands (cm^–1^)			
AECO	WECO	AESC	description of functional groups
3904		3906	
3820	3822	3822	
3747	3749	3753	
3431		3424	O–H of intermolecular hydrogen bond
3049		3050	OH–N (OH forms a hydrogen bond with the NH bond of the adjacent molecule)
		3012	CH stretch in aromatics
2923	2923	2925	CH_3_ aliphatic stretch
2852	2853	2849	CH_2_ aliphatic stretch
	2732	2822	CHO stretch of RHCO out of a plane in aldehydes
		2689	(O=)PO–H in phosphonic acid
		2664	(O=)PO–H in phosphonic acid
		2410	P–H in phosphine
		2404	P–H in phosphine
2362		2378	P–H in phosphine
	2346	2345	
		2259	C≡C of alkynes (very weak (often indistinguishable))
		2098	N=C in R–NCS
		1800	C=O stretch of R–CO–Cl in acid chloride
1695	1699	hide	C=O stretch
1605	1604	1628	amide NH out of plane in RCONH_2_
1457	1462	1447	methyl –CH_3_ group
1376	1377	1272	–CH_2_– and CH_3_ of RCH_2_CH_3_ in alkanes
1133		1100	P–H bending
1033	1027	1030	sulfoxide C_2_S=O functional group
865	881	860	aromatic C–H out-of-plane deformation of four adjacent hydrogen atoms
		827	=CH out of a plane
809	810	805	aromatic C–H out-of-plane deformation of a single adjacent hydrogen atom
755		750	4 hydrogen atoms adjacent to an aromatic ring
	724	720	alkyl chains longer than 4 methylene units
621	615	620	#CH bending vibration of RC#CH in alkynes
545		550	C–Cl stretch of R–Cl in alkyl halides
530, 514		530, 515	C–Br stretch of R–Br in alkyl halides

As mentioned before, the asphaltene samples obtained from solid deposits before the choke have a much more complicated structure than the asphaltene sample extracted from crude oil. This complicated process may be due to the simultaneous precipitation of asphaltene and wax from the bottom hole to the surface by a drop in pressure and temperature, well stimulation operation, and the addition of some acidic components of crude oil to the solid deposits. In conclusion, this phenomenon indicates that the remedies for removing the solid deposits in the annulus, choke, and pipelines must be stronger than that of bottom-hole asphaltene or precipitated asphaltene in the reservoir.

In these experiments, xylene, kerosene, and gasoline, as the most widely available aromatic and aliphatic solvents, were used to retrieve the damaged permeability due to asphaltene deposition in cores. In addition, the equivalent injection rates of 1 and 3 bbl/min were used to investigate the effect of injection rate on solvent performance. The retention time, pressure, and temperature of 2 days, 4000 psi, and 80 °C, respectively, were used constantly.

**Figure 8:**
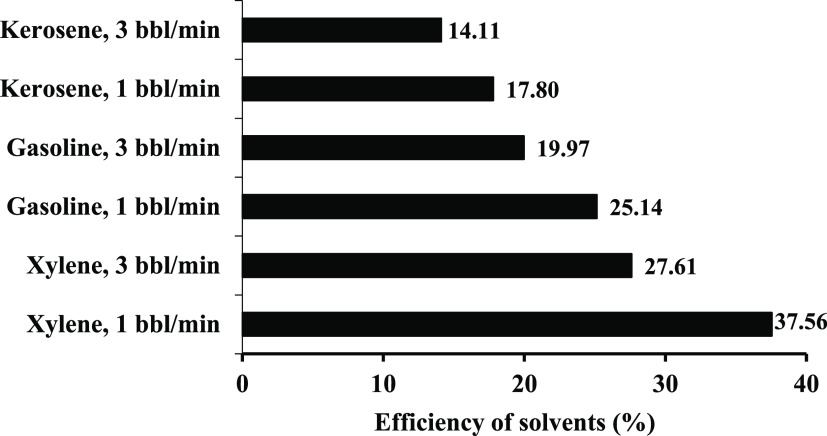
Efficiency of xylene, gasoline, and kerosene in reversing the damaged permeability.

The experimental results showed that permeability is reduced because of asphaltene deposition inside the cores and some fraction of lost permeability is recoverable by solvent injection, as presented in Table 8. Among the tested solvents, xylene, gasoline, and finally kerosene have the highest efficiency in dissolving the deposited asphaltene and improving the permeability, as presented in Figure 8. In addition, the injection rate also influenced the efficiency of the solvents. Increasing the injection rate from 1 to 3 bbl/min decreased the efficiency of all solvents. For instance, xylene with a 1 bbl/min injection rate resulted in an efficiency of 37.56%, and the value was reduced to 27.61% when the injection rate increased to 3 bbl/min. One observation from experimental results was that core flood experiments also confirmed static tests as xylene performed than gasoline and kerosene. As the most efficient aromatic solvent, xylene performed well in dissolving asphaltene and retrieving the damaged permeability. In addition, the aromatic fractions of the gasoline resulted in a better gasoline performance than kerosene. Another observation from the results was that the injection rate effectively dissolved the asphaltene deposits and retrieved the damaged permeability. The injection rate of 1 bbl/min for the three tested solvents always resulted in higher efficiency than 3 bbl/min. Increasing the injection rate increases the velocity of the injected solvent in the porous media, thus reducing the efficient contact time between the deposited asphaltene particles and solvent. This will result in reduced efficiency with a high injection rate. This is very important in the field application of solvent flooding with coiled tubing, where the injection rate affects the efficiency of the injected solvent.

**Table 8:** Effect of Different Solvents with Low and High Injection Rates on the Permeability

solvent	equivalent injection rate (bbl/min)	initial permeability (mD)	permeability after damage (mD)	permeability after solvent injection (mD)	damage due to asphaltene deposition (%)
xylene	1	5.83	1.12	2.19	81
xylene	3	7.28	1.31	2.01	82
kerosene	1	9.83	1.67	1.75	83
kerosene	3	11.48	1.59	1.62	86
gasoline	1	7.12	1.24	1.79	83
gasoline	3	7.71	1.32	1.54	83

#### 3.3.4 Effect of Mixed Solvents on Permeability

In this section, the effects of mixed solvents in retrieving the damaged permeability were investigated. In this regard, different solvents were prepared by 50 vol % of xylene, kerosene, and gasoline and 50 vol % of gas condensate. Each solvent was injected by equivalent flow rates of 1 and 3 bbl/min. In addition, a retention time of 2 days, a pressure of 4000 psi, and a temperature of 80 °C were applied accordingly. Table 9 presents the experimental results achieved in this section.

**Table 9:** Effect of Mixed Solvents with Low and High Injection Rates on the Permeability

solvent	equivalent injection rate (bbl/min)	initial permeability (mD)	permeability after damage (mD)	permeability after solvent injection (mD)	damage due to asphaltene deposition (%)	efficiency of the solvent (%)
xylene and gas condensate	1	5.83	1.12	2.19	81	29.68
xylene and gas condensate	3	7.28	1.31	2.01	82	22.42
kerosene and gas condensate	1	9.83	1.67	1.75	83	13.87
kerosene and gas condensate	3	11.48	1.59	1.62	86	8.71
gasoline and gas condensate	1	7.12	1.24	1.79	83	17.64
gasoline and gas condensate	3	7.71	1.32	1.54	83	13.57

The injected solvents were able to recover some fraction of damaged permeability. Among the tested solvents, a mixture of xylene/gas condensate performed better than a mixture of gasoline/gas condensate and kerosene/gas condensate. In addition, solvents with an injection rate of 1 bbl/min performed better than those of 3 bbl/min. The effective contact of solvents with the deposited asphaltene decreases with increasing injection rate, thus decreasing the effectiveness of the solvent in dissolving the deposited asphaltene. Another observation from the results is that the efficiency of mixed solvents is significantly lower than that of pure solvents alone. It seems that adding gas condensate to solvents lowered the efficiency of solvents. For instance, xylene with an injection rate of 1 bbl/min resulted in an efficiency of 37.56%, and the value was decreased to 29.68% when a mixture of gas condensate and xylene was used. The same behavior is observed for the other solvents as well. The static tests showed that gas condensate did not affect asphaltene and could only dissolve wax. In addition, the WAT of the crude oil used in this study is about 64 °C, whereas the operating temperature is 80 °C. This suggests that only asphaltene is deposited inside the pores, and no wax is deposited. Thus, not only the injection of a mixture of solvents with gas condensate has no additional effect on improving the permeability but it also hinders the solvents’ efficiency. The volume of solvents (xylene, gasoline, and kerosene) is decreased when mixed with gas condensate; thus, the efficiency is reduced as a result of these experiments.

## 4 Conclusions

The experimental investigations yielded several quantitative and qualitative assertions about solvent use in reversing permeability damage and removing deposited solids in oil well settings. The asphaltene samples obtained from solid deposits behind the surface of the choke have a much more complicated structure than that of the asphaltene sample extracted from crude oil. Moreover, based on the WAT of the crude oil (64 °C) and the reservoir temperature (∼80 °C), it is deemed that only asphaltene deposition occurs inside the reservoir, while wax deposition occurs inside the tubing string and at the surface facilities. Hence, the screening of the solvents should be in a way to address these issues, wherein the remedies for removing the solid deposits in the annulus, choke, and pipelines must be stronger than bottom-hole asphaltene or precipitated asphaltene in the reservoir.

The static tests showed that xylene, toluene, gasoline, kerosene, and gas condensate could dissolve solid deposits to some extent; however, diesel and gas condensate were ineffective in dissolving the solid deposits due to the formation of new bonds that increase the weight of asphaltene. Increasing the retention time and volume of solvent during static tests effectively increased solvents’ efficiency in dissolving solid deposits and asphaltene, but the magnitude of the effects was solvent-dependent. In the core flooding experiments, to reverse the 79–91% of core permeability loss by asphaltene deposition, the maximum efficiency (obtained at 1 bbl/min rather than 3 bbl/min) of xylene, gasoline, and kerosene to restore the damage permeability was 37.56, 25.14, and 17.80%, respectively.

For cleaning the surface facilities and tubing string, where asphaltene and wax depositions exist, solvents alone or their mixture with gas condensate could be very effective in dissolving the solid deposits. For this purpose, xylene, toluene, gasoline, and kerosene performed very well in dissolving the solid deposits. In addition, gas condensate and xylene mixture, as well as mixtures of gas condensate and gasoline, resulted in reasonable efficiency in dissolving the solid deposits. In contrast, for the permeability reduction inside the porous media of the reservoir because of asphaltene deposition and in the absence of wax deposition, the selection of suitable solvents is slightly different. Xylene, gasoline, and kerosene effectively dissolved asphaltene and restored the damaged permeability due to asphaltene deposition.

## Supporting Information Available

Compositions of the reservoir fluids (Table S1), XRD image of the dolomite rock sample and ζ-potential values (Figure S1), schematic diagram of the wax and asphaltene extraction process (Figure S2), physical properties of the core samples used in this study (Table S2), calculated injection rates (Table S3), schematic diagram of the core flood system for dynamic tests (Figure S3), and the summary of the core flooding experiments (Figure S4) (PDF)

## Abbreviations

AECO: asphaltene extracted from crude oil
AESC: asphaltene extracted from surface choke
FTIR: Fourier transform infrared spectroscopy
HPLC: high-performance liquid chromatography
mD: milli-Darcy
mV: millivolts
ppm: part per million
PV: pore volume
PZC: point of zero charge
SC: surface choke
TDS: total dissolved solids
WAT: wax appearing temperature
WECO: wax extracted from crude oil
XRD: X-ray diffraction
