# Supplementary material for: Comparative Static and Dynamic Analyses of Solvents for Removal of Asphaltene and Wax Deposits above- and below-Surface at an Iranian Carbonate Oil Field
Source: ACS Omega. 2023 Jul 6;8(28):25525–37. doi: 10.1021/acsomega.3c03149 (PMC10357422; doi:10.1021/acsomega.3c03149)
Supplement: Supplementary file 1 — ao3c03149_si_001.pdf [file ao3c03149_si_001.pdf]

Supporting Information

Comparative static and dynamic analyses of solvents for removal of asphaltene and wax deposits above- and below-surface at an Iranian carbonate oil field

Milad Norouzpour[1], Amin Azdarpour[1, *], Rafael M. Santos[2, *], Ali Esfandiarian[1], Moein Nabipour[1], Erfan Mohammadian[3], Abbas Khaksar Manshad[4], Alireza Keshavarz[5,6, *]

[1]Department of Petroleum Engineering, Marvdasht Branch, Islamic Azad University, Marvdasht, Iran.

[2]School of Engineering, University of Guelph, Guelph, N1G 2W1, Ontario, Canada.

[3]Key Laboratory of Continental Shale Hydrocarbon Accumulation and Efficient Development, Northeast Petroleum University, Daqing, Heilongjiang 163318, China.

[4]Department of Petroleum Engineering, Abadan Faculty of Petroleum Engineering, Petroleum University of Technology (PUT), Abadan, Iran.

[5]Petroleum Engineering Discipline, School of Engineering, Edith Cowan University, 270 Joondalup Dr, Joondalup 6027, WA, Australia.

[6]Centre for Sustainable Energy and Resources, Edith Cowan University, Joondalup, WA 6027, Australia.

[*]Corresponding author: amin.azhdarpour@iau.ac.ir, santosr@uogueph.ca, a.keshavarz@ecu.edu.au

Table S1. Composition of crude oil used in this study.

Components	Dead oil (mol %)	Associated Gas (mol %)	Reservoir fluid (mol %)	Reservoir fluid (g/mol)
H_2S	0.00	0.55	0.33	34.1
N_2	0.00	2.53	1.52	28.0
CO_2	0.00	3.37	2.03	44.0
C_1	0.00	80.79	48.68	16.0
C_2	0.00	7.95	4.79	30.1
C_3	0.00	2.90	1.75	44.1
iC_4	0.00	0.46	0.28	58.1
nC_4	0.00	0.80	0.48	58.1
iC_5	0.00	0.20	0.12	72.2
nC_5	0.00	0.19	0.11	72.2
C_6	6.62	0.06	2.67	84.0
C_7	13.16	0.21	5.36	96.0
C_8	11.88	0.00	4.72	107
C_9	11.55	0.00	4.59	121
C_{10}	4.68	0.00	1.86	134
C_{11}	2.95	0.00	1.17	147
C_{12+}	49.16	0	19.54	365.7
**MW of Heavy Component**	**365.74**	**g/mol**		
**Specific Gravity**	**0.9223**			
**MW of Reservoir oil**	**105.71**	**g/mol**		
**MW of Dead oil**	**235.29**	**g/mol**		

(a)

(b)

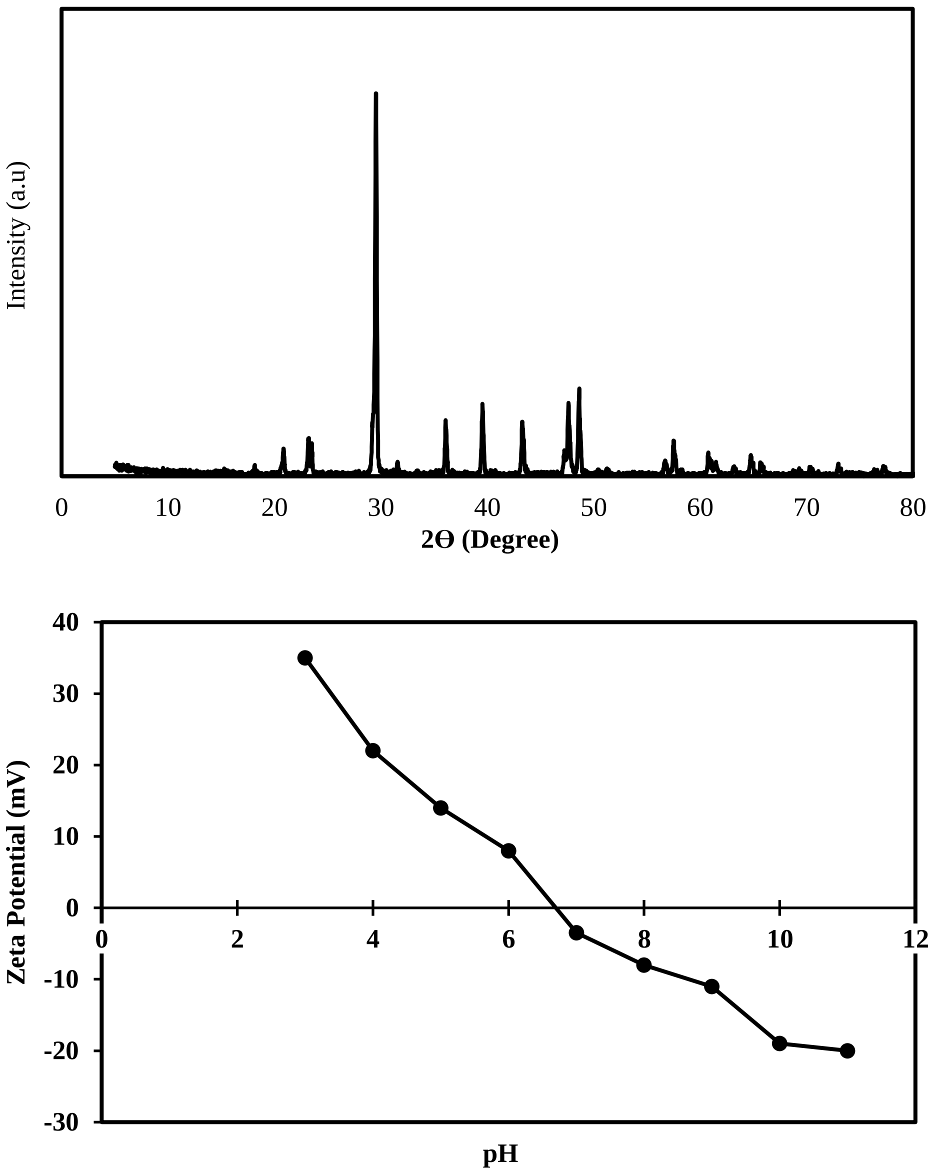

Fig. S1. a) XRD pattern of dolomite sample; b) variation of zeta potential with equilibrium pH of dolomite powder suspended in the water phase.

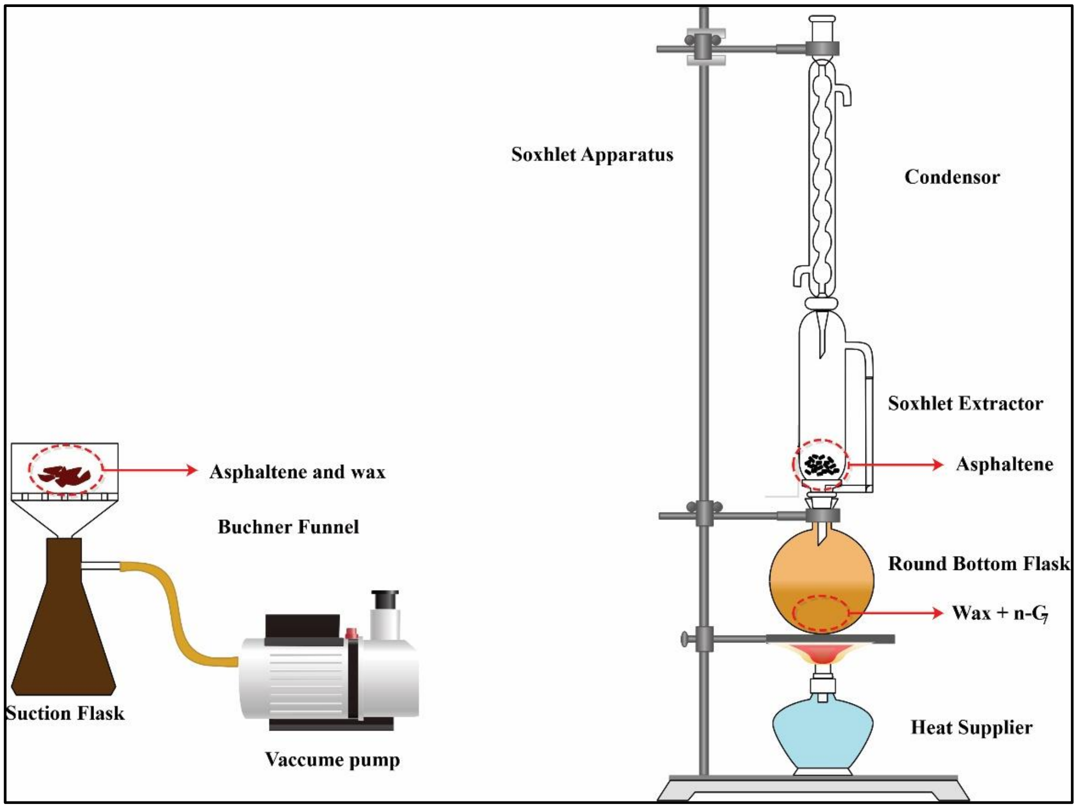

Fig. S2. Schematic diagram of wax and asphaltene extraction of crude oil.

Table S2. Physical characteristics of core samples used in this study.

Sample name	Porosity (%)	Permeability (mD)	Length (cm)
S1	12.74	6.83	6.73
S2	14.29	8.19	6.75
S3	11.38	5.83	6.79
S4	15.74	7.28	6.80
S5	15.39	7.12	6.73
S6	13.75	7.71	6.78
S7	15.29	9.83	6.78
S8	16.17	11.48	6.80
S9	14.84	7.11	6.80
S10	12.46	6.29	6.80
S11	15.92	6.18	6.76
S12	13.73	8.18	6.78
S13	12.49	8.58	6.79
S14	14.83	11.83	6.78

Table S3. Summary of calculated injection rates to cores.

Sample name	Injection rate to the reservoir (bbl/min)	Equivalent injection rate to cores (cm^3/min)
S1	1	1.68
	3	5.05
S2	1	1.50
	3	4.50
S3	1	1.88
	3	5.65
S4	1	1.39
	3	4.09
S5	1	1.39
	3	4.18
S6	1	1.56
	3	4.68
S7	1	1.40
	3	4.21
S8	1	1.33
	3	3.98
S9	1	1.45
	3	4.34
S10	1	1.72
	3	5.16
S11	1	1.35
	3	4.04
S12	1	1.56
	3	4.69
S13	1	1.72
	3	5.15
S14	1	1.45
	3	4.34

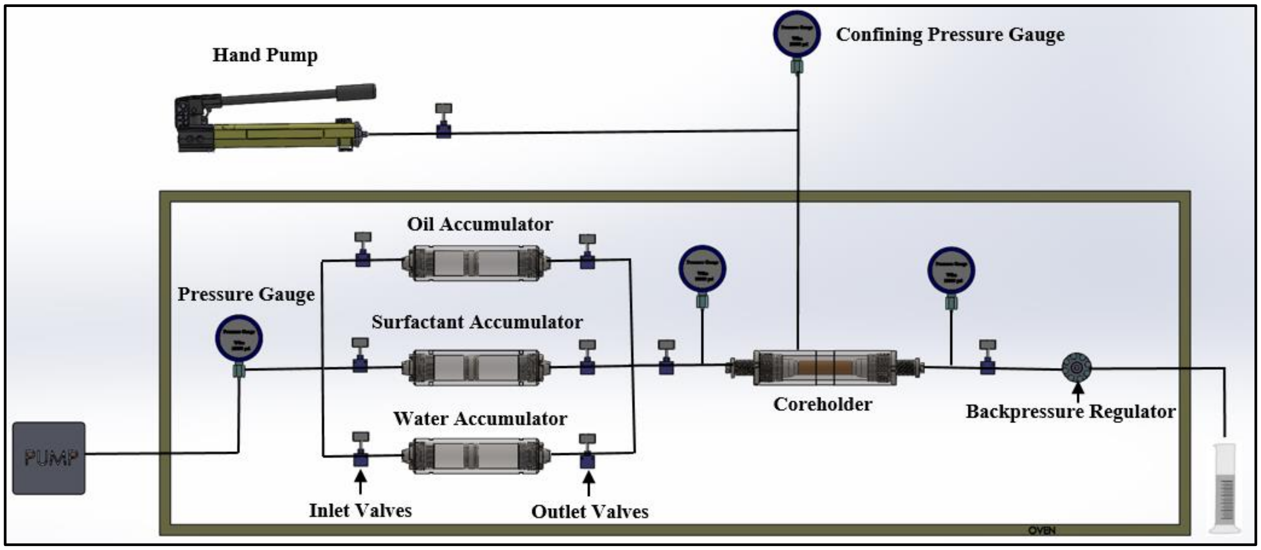

Fig. S3. Schematic of the core flood system utilized for dynamic tests in this study (Norouzpour et al., 2022). Re-used with permission from Elsevier (5545491258520).

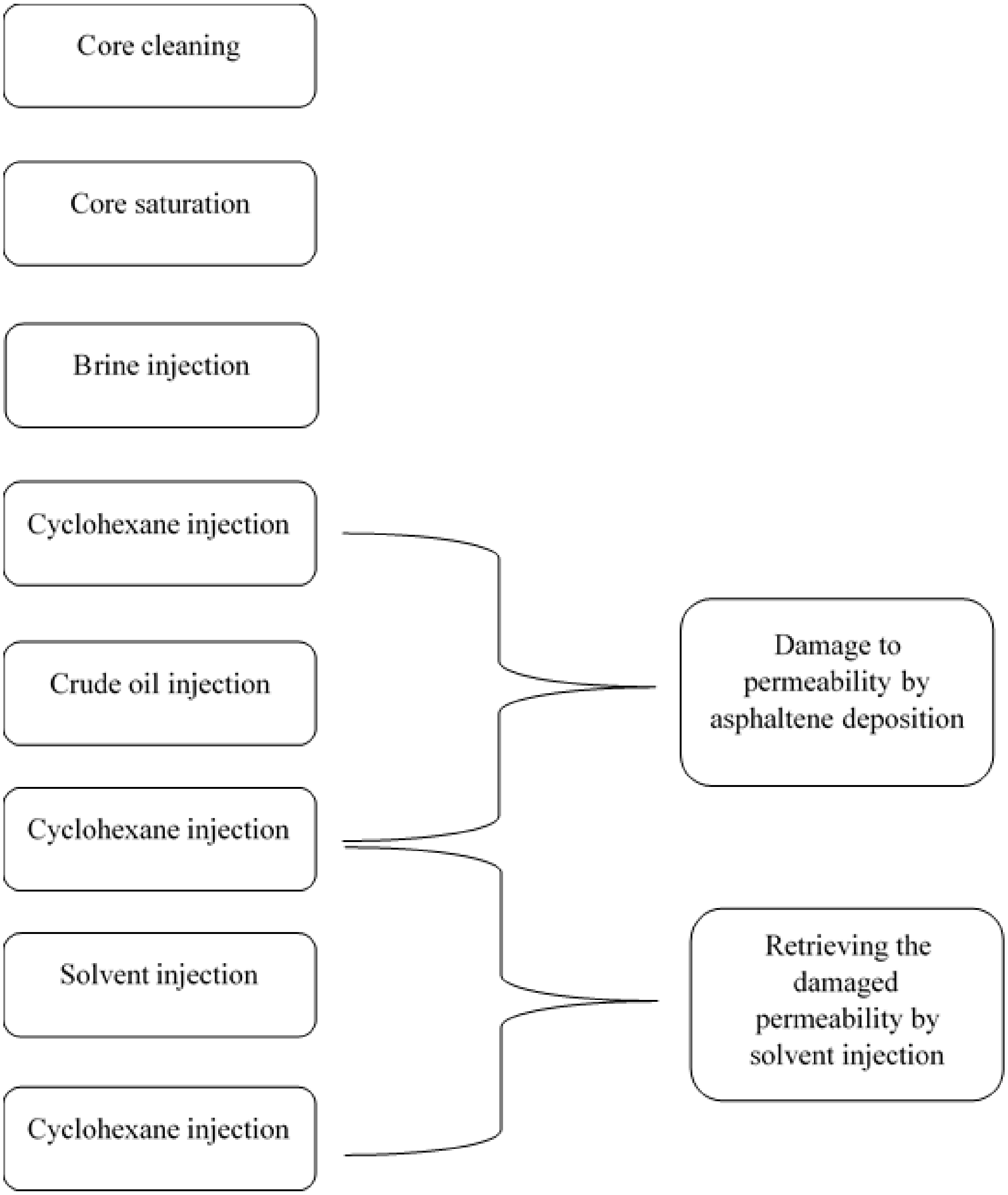

Fig. S4. Summary of core flood experiments.
